# The effect of subjective age on loneliness in the old adults: The chain mediating role of resilience and self-esteem

**DOI:** 10.3389/fpubh.2022.907934

**Published:** 2022-08-02

**Authors:** Jin Xie, Bo Zhang, Zhendong Yao, Wenya Zhang, Jingli Wang, Chun-ni Zhao, Xinquan Huang

**Affiliations:** ^1^Mental Health Service Center, Huanghuai University, Zhumadian, China; ^2^School of Educational Sciences, Hunan Normal University, Changsha, China; ^3^School of International Education, Huanghuai University, Zhumadian, China; ^4^Normal College, Hunan University of Arts and Science, Changde, China; ^5^Counseling Center of Zhumadian Psychiatric Hospital, Zhumadian, China; ^6^School of Marxism, Foshan University, Foshan, China; ^7^School of Marxism, Guizhou Medical University, Guiyang, China

**Keywords:** subjective age, loneliness, resilience, self-esteem, old adults

## Abstract

**Objectives:**

This study aimed to explore the effect of subjective age on loneliness in old adults, and the mediating role of resilience and self-esteem in subjective age and loneliness.

**Methods:**

Approximately 450 old adults from Jiangxi, Hunan, Henan provinces completed the third edition of the Loneliness Scale (UCLA-LS III), Age Decade Scale (ADS), Connor-Davidson Resilience Scale (CD-RISC), and Self-Esteem Scale (SES).

**Results:**

(1) Subjective age was significantly positively correlated with loneliness. (2) Resilience, self-esteem, and loneliness were significantly negatively correlated. (3) Subjective age affected loneliness through the mediating effects of resilience and self-esteem, respectively. (4) Resilience and self-esteem played a chain mediating role between subjective age and loneliness.

**Conclusion:**

Resilience and self-esteem can directly affect the loneliness of the old adults alone and can also affect the loneliness of the old adults through the chain mediating effect of resilience and self-esteem.

## Introduction

Loneliness, a negative emotional experience, is pervasive in human beings. Loneliness is one of the most direct negative manifestations when an individual's communication needs are not met. It is caused by the difference between the individual's expectation of social communication and the actual level of social communication (1). Studies have found that loneliness was an important risk factor for many negative life outcomes (2), it not only damages the individual's mental health and increases the risks of depression and anxiety (3) but also has a certain impact on the individual's physiological and cognitive abilities, increasing sleep disorder and the risk of cancer, cardiovascular and cerebrovascular diseases, stroke, and other diseases and reducing the body's immune ability and the individual's cognitive functions such as processing speed, immediate recall, and delayed recall (4–6). According to a cross-sectional survey, a ratio of 20.1% to 78.1% of the old adults in the world was lonely to different degrees, and their loneliness experiences were obvious (7). Previous studies have pointed out that in old age, part of the old adults who were not lonely would turn into mild loneliness, and old age was the high incidence period of increase of the number of loneliness and the aggravation of loneliness (8). Studies have also pointed out that in old age, due to the physical and psychological constraints of old adults, they were forced to reduce their social network, thus reducing their social relationship satisfaction and aggravating the degree of loneliness (9). Studies have shown that there were significant gender differences in loneliness among old adults, and women showed stronger loneliness than men (10). In terms of age, loneliness was more prevalent among old adults, especially those who were very old, according to the survey (11). The degree of loneliness varied at different ages, with a peak at the age of 20, 50, and 80 years, and a high incidence of loneliness at the early adult stage, the late middle age stage, and the old age stage (12).

Subjective age is an individual's self-perceived age, a self-assessment of one's position in the course of life, and a favorable indicator of aging (13). Generally speaking, when the subjective age of the old adult was younger than the chronological age (i.e., the age in the general sense arranged in time series), it reflected the positive bias of the subjective age, and when the subjective age was older than the chronological age, it reflected the negative bias of the subjective age. The subjective age of the old adults showed an overall decreasing trend with time, in which the change of perceived age was not obvious, and the gap between apparent age and chronological age narrowed over time (14). A study on the relationship between loneliness and neurological symptoms in old adults suggested that subjective age can potentially alleviate the adverse effects of loneliness on neurological symptoms (15). At the same time, subjective age, as a regulating mechanism of aging, can have a certain impact on loneliness by regulating aging. Studies have shown that the more negative the self-perceived aging attitude, the higher the degree of loneliness (16). The daily fluctuation of subjective age of the old adults was significantly correlated with emotional bias: the older subjective age was more likely to produce negative emotions, while the younger subjective age was able to prevent the production of negative emotions (17, 18).

Resilience refers to an individual's ability to recover from a heavy shock or exposure to pressure (19). Previous studies have shown that individuals with high resilience can adjust their self-state in time to adapt to the environment after encountering stressful events and show less depression and anxiety than individuals with low resilience (20). Individuals with a high level of psychological resilience often have qualities such as optimism and tenacity, and they can actively mobilize strong inner strength and adopt adaptive and coping strategies to relieve anxiety when confronted with emergencies such as COVID-19. For example, a study investigated the mental health status of 3,042 subjects during the COVID-19 epidemic, and the results suggested that resilience can relieve the stress caused by the pandemic (21). Studies have also shown that individuals with strong psychological resilience have the qualities of optimism, perseverance, and positive coping, which are conducive to actively dealing with psychological distress and better adapting to society (22).

Self-esteem refers to an individual's emotional experience of self-worth and self-ability, which is an emotional component with certain evaluative significance in the self-evaluation system. Self-esteem plays an important role in emotion and social adaptation. The research showed that self-esteem and loneliness were significantly negatively correlated with predictability (23, 24). The social cognitive theory also suggested that individuals with low self-esteem engaged in certain behavioral and cognitive processes that hinder the development of social relationships, thereby increasing loneliness (25), which may in turn weaken self-esteem (26). Therefore, self-esteem was not only the antecedent of loneliness but also the consequence of loneliness, and in this mutual process, self-esteem and loneliness strengthened each other over time (27). To further understand the relationship between self-esteem and loneliness, some studies have also studied the potential mechanism between loneliness and self-esteem. The results showed that social anxiety played a moderate mediating role between self-esteem and loneliness (28).

Based on the above literature review, the following hypotheses were proposed in this study:

**Hypothesis 1**: The subjective age of the old adults is positively correlated with loneliness, while the resilience and self-esteem of the old adults are negatively correlated with loneliness. As a self-regulating mechanism of age, subjective age can detect an old adult's self-attitude. Loneliness, as a negative emotion, subjective age can also affect loneliness by regulating emotional health. The higher the level of resilience of the old adults, the more self-adjustment they can make, and the lower their loneliness will be. The lower the level of resilience of the old adults, the more they cannot adjust themselves, and the higher the degree of loneliness. Low self-esteem will affect the behavior and cognitive process of the old adults, hinder the development of social relations, and thus increase loneliness. A high level of self-esteem is conducive to enhancing the sense of self-worth of the old adults, promoting the development of social relations, and thus reducing loneliness.

**Hypothesis 2**: Subjective age can affect loneliness in old adults through the mediating role of resilience. When faced with loneliness, the old adults with a high level of resilience can fully mobilize all kinds of resources, including the estimation of subjective age, to deal with it, thus reducing the level of loneliness, while the old adults with a low level of resilience choose to escape and retreat to deal with it, thus increasing loneliness.

**Hypothesis 3**: Subjective age can affect loneliness in old adults through the mediating role of self-esteem. Self-esteem is a subjective measure of the interpersonal relationship between old adults and others. The old adults with higher self-esteem show lower loneliness, which will affect the formation of self-esteem in the old adults.

**Hypothesis 4:** Subjective age can affect loneliness in old adults through the chain mediating role of resilience and self-esteem. On the basis of hypothesis 2 and hypothesis 3, hypothesis 4 was proposed.

**Figure 1:**
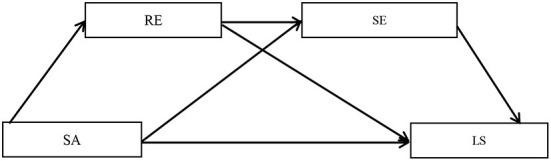
A chain mediation model of resilience and self-esteem in the relationship between subject age and loneliness. SA, subject age; LS, loneliness; RE, resilience; SE, self-esteem.

The detailed hypothetical model is shown in Figure 1.

## Methods

### Participants

A convenient sampling method was used to conduct a questionnaire survey on 450 old adults from Jiangxi, Hunan, Henan provinces. First of all, five postgraduates who majored in psychology were trained as examiners, so that they could be familiar with the matters needing attention in the questionnaire survey. Then, the questionnaire survey was conducted in batches and collectively. After the survey, the questionnaires were collected on the spot. A total of 450 questionnaires were distributed, 10 invalid questionnaires were excluded, and 440 valid questionnaires were obtained, with a ratio of 97.8%. The specific information of the samples is shown in Table 1.

**Table 1:** Sample details.

**Variable**	**Characteristics**	**Number**	**Percentage (%)**
Gender	Male	130	29.5
	Female	310	70.5
Age group	60–69 years old	220	50.0
	70–79 years old	168	38.1
	Over 80 years old	52	11.9
Education level	Junior high school and below	185	42.0
	Senior high school	168	38.2
	University and above	87	19.8
Living status	Living with offspring	60	13.6
	Living With Spouse	245	55.7
	Living with offspring and spouse	109	24.8
	Living alone	26	5.9
Health condition	Poor	60	13.6
	Ordinary	268	60.9
	Well	112	25.5

### Measures

#### Loneliness scale (UCLA-LS III)

The third edition of the Loneliness Scale (UCLA-LS III) was compiled by Russell (29). This study has shown that the scale had good reliability and validity (31). The scale in this study included 20 items, such as “Do you often feel that you have a lot in common with people around you?” Four points were used to score, 1 means “never,” 2 means “very few,” 3 means “sometimes,” and 4 means “always.” The higher the total score, the higher the degree of loneliness. In this study, Cronbach's α of the whole scale was 0.869.

#### Subjective age scale

The subjective age was measured by the age group scale which was developed by Barak and Schiffman (30). The scale consisted of four questions. The subjects only needed to fill in the numbers on the blank line according to their actual situation. The topics were: (1) I feel like I am only __ years old. (This item measured the perceived age of the individual, that is, the age perceived by the individual himself). (2) I look like I am only __ years old. (Appearance age was measured, that is, the individual perceived age on their own appearance). (3) When working and doing things, I feel like I am only __ years old. (Behavioral age was measured, that is, the age perceived by individuals based on the activities they engaged in). (4) I have the same hobbies as people at __. (Interest age was measured, that is, the age perceived by individuals based on their own interests). The age of the four measured dimensions was subtracted from the actual age, and the average was taken as the difference between the subjective age and the actual age, which was used in subsequent calculations. Since the scale was widely used abroad, but less used in China, confirmatory factor analysis was carried out in this study to explore the applicability of this scale in old adults. The confirmatory factor analysis indexes were as follows: χ^2^/df = 1.472, *P* < 0.001, GFI = 0.935, comparative fit index (CFI) = 0.996, root-mean-square error of approximation (RMSEA) = 0.033, and standardized root-mean-square residual (SRMR) = 0.016. All the indexes were in line with the requirements of surveying. In this study, Cronbach's α of the whole scale was 0.870.

#### Connor-Davidson resilience scale

The resilience scale was adopted to measure the resilience of the old adults (31). Previous studies have shown that the scale had good reliability and validity (32). There were 25 items (such as “I always can recover from the setbacks quickly.”) on the scale, which were 5-point Likert scale ranging from “1 = completely incorrect” to “5 = almost always.” The higher the scale score, the higher the level of resilience. The Cronbach's α coefficient of the scale in this study was 0.96.

#### The self-esteem scale

The SES was adopted to measure the self-esteem of old adults (33). There were 10 items (such as “I have a positive attitude toward myself.”) on the scale, which was a 4-point Likert scale ranging from “1 = Very consistent” to “4 = Very inconsistent.” The higher the scale score, the higher the level of self-esteem. Previous studies have shown that the scale has good reliability and validity (34). The Cronbach's α coefficient of the scale in this study was 0.844.

### Procedures

In this study, group testing was adopted, and interviewers who majored in psychology administered the test. The interviewers distributed questionnaires and read instructions to the subjects. The questionnaires were collected after the subjects completed the questions.

### Data analysis

In this study, SPSS 22.0 were used to manage and analyze the data. Among them, descriptive statistics and correlation analysis were completed. Among them, the SPSS process component compiled by Hayes was used to test the mediation model (35). Besides, AMOS23.0 was used to construct the structural equation modeling in this study.

## Results

### Common method bias test

Harman's one-factor test for common method deviation was used, and the statistical results showed that there were 19 factors with eigenvalues > 1. The first factor can explain 17.05% of the variance, which was < 40% of the critical criterion (36). Therefore, there was no common method deviation in the data of this study.

### Descriptive statistics and correlation analysis of each variable

Correlation analysis was carried out on resilience, subjective age, self-esteem, and loneliness, and it was found that the old adults' loneliness was significantly positively correlated with subjective age. Loneliness in the old adults was significantly negatively correlated with resilience and self-esteem. The subjective age of the old adults was significantly negatively correlated with resilience and self-esteem. There was a significantly positive correlation between self-esteem and resilience in the old adults, as shown in Table 2.

**Table 2:** Descriptive statistics and correlation analysis of each variable.

	**M**	**SD**	**SA**	**LS**	**RE**	**SE**
SA	9.11	6.61	1			
LS	40.60	9.13	0.31**	1		
RE	27.11	5.72	−0.30**	−0.52**	1	
SE	30.94	3.95	−0.29**	−0.50**	0.33**	1

**p < 0.01.

*SA, subject age; LS, loneliness; RE, resilience; SE, self-esteem*.

### Structural equation modeling of the chain mediation effect

**Figure 2:**
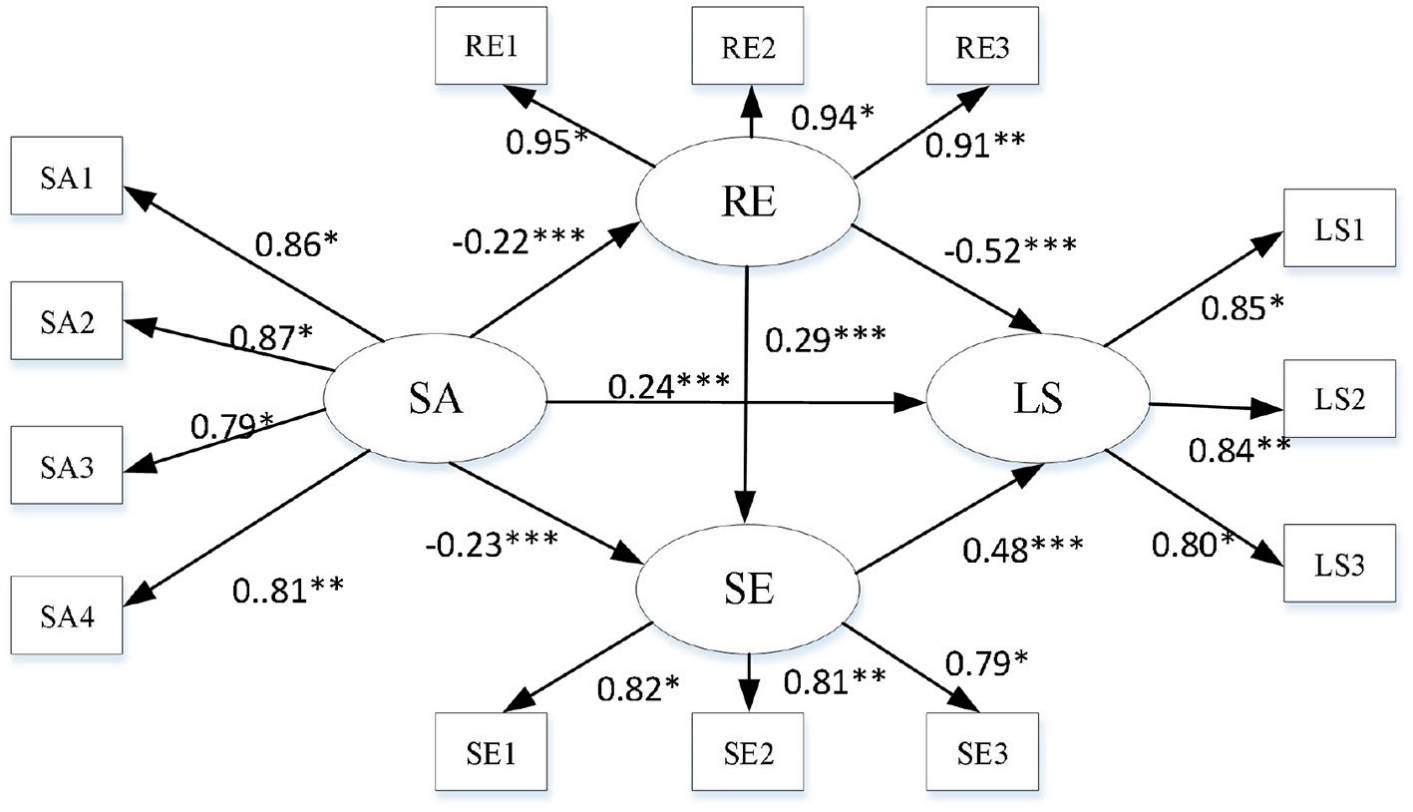
A chain mediating model of subjective age predicting the loneliness in the old adults. SA, subject age; LS, loneliness; RE, resilience; SE, self-esteem. *p < 0.05; **p < 0.01; ***p < 0.001.

Based on the hypothetical model and the analysis of the relationship between various variables, a chained mediation structural equation model with latent variables was established to investigate the mediating effect of subjective age, resilience, self-esteem, and loneliness among old adults. The results show that the fitting index of the model is: χ^2^ = 427.00, χ^2^/df = 7.12, NFI = 0.94, CFI = 0.95, IFI = 0.95, RMSEA = 0.08, and the fitting is good (refer to Figure 2).

The results showed that subjective age negatively predicted resilience (β = −0.22, *t* = −4.75, *p* < 0.001), significantly negatively predicted self-esteem (β = −0.23, *t* = −4.84, *p* < 0.001), and significantly positively predicted loneliness (β = 0.24, T). Resilience can significantly positively predict self-esteem (β = 0.29, *t* = 6.27, *p* < 0.001) and negatively predict loneliness (β = −0.52, *t* = −12.11, *p* < 0.001).

### Significant test and effect analysis of mediating effect of resilience and self-esteem

Bootstrap analysis was used to test the significance of the mediating effect of the model. The results showed that resilience and self-esteem were the mediating variables of the influence of subjective age on loneliness in old adults, and the total effect size (95% CI) of the mediating effect was 0.23 (0.16–0.40), accounting for 70.76% of the total effect. Subjective age can not only directly affect the loneliness of the old adults but also indirectly affect the loneliness of the old adults through resilience and self-esteem, and resilience and self-esteem played a mediating role. That is, the mediating effect of resilience and self-esteem can be achieved through the following three paths (Table 3): (1) Path 1 (0.12): subject age → resilience → loneliness; Path 2 (0.08): subject age → self-esteem → loneliness; Path 3 (0.02): subject age → resilience → self-esteem → loneliness, which accounted for 35.18, 24.29, and 9.46% of the total effect, respectively.

**Table 3:** Bootstrap analysis of the mediating effect of resilience and self-esteem between subjective age and loneliness.

	**Indirect effect value**	**Boot SE**	**Boot CI lower**	**Boot CI upper**	**Relative mediation effect**
Total mediation effect	0.23	0.04	0.15	0.31	70.76%
Path 1: SA → RE → LS	0.12	0.03	0.66	0.18	35.18%
Path 2: SA → SE → LS	0.08	0.03	0.03	0.14	24.29%
Path 3: SA → RE → SE → LS	0.02	0.01	0.01	0.04	9.46%

*SA, subject age; LS, loneliness; RE, resilience; SE, self-esteem*.

## Discussion

### The mediating roles of resilience and self-esteem in subjective age and loneliness

The results of the correlation analysis between subjective age and loneliness showed that subjective age was significantly positively correlated with loneliness, which verified the first half of Hypothesis 1. Loneliness implied the objective lack of social relations (37), and the older or younger subjective age reflected the dynamic interaction between the individual's own cognition, the internal standard development model, and the external signs at a specific stage of life (38). Specifically, the objective loss in the old age stage was often caused by aging, and there were differences in aging among individuals due to the influence of physiological, psychological, and environmental factors. Subjective age can reflect the status of self-development that changed with the life cycle. Subjective age can reflect an aging attitude, and a positive aging attitude can effectively reduce the impact of objective loss, while a negative aging attitude can aggravate the impact of objective loss. Therefore, subjective age was significantly positively correlated with loneliness. In this study, the results of the correlation analysis between resilience and loneliness showed that resilience and loneliness were significantly negatively correlated. The results indicated that the higher the degree of resilience in the old adults, the lower the degree of loneliness, which verifies the second half of Hypothesis 1. Good resilience will have a positive impact on an individual's mental health, such as eliminating negative emotions and loneliness. In old age, a series of feeling of loss caused by aging makes the old adults actively shrink their social network, thereby making the problem of loneliness more serious. At the same time, in old age, the old adults showed stronger emotional needs, which changed the resilience of the old adults, and this series of changes made the relationship between loneliness and resilience closer. The results of the correlation analysis between self-esteem and loneliness in this study showed that self-esteem and loneliness were significantly negatively correlated. This showed that the higher the level of self-esteem of the old adults, the lower the degree of loneliness, and the lower the level of self-esteem, the higher the degree of loneliness, which verified the second half of Hypothesis 1. According to the social gauge theory of self-esteem, self-esteem can reflect the emotional state of an individual's integration into the interpersonal relationship, that is, self-esteem can reflect whether an individual has a good interpersonal relationship, thus reflecting emotional experience (39). Moreover, some studies have explained the relationship between self-esteem and loneliness from a cognitive perspective (40). According to cognitive theory, individuals with low self-esteem exhibited more internalized symptoms and had dysfunctional self-schemas in their memory, leading to the process of self-related information. The low self-esteem of the old adults led to negative cognition of the relationship between self and others, which was handled in a negative way, and finally led to loneliness.

### The independent mediating role of resilience between subjective age and loneliness

The results of the mediating effect test in this study showed that resilience was an important mediator between subjective age and loneliness; therefore, Hypothesis 2 was verified. Subjective age can reflect the attitude toward aging, and young subjective age made the old adults face aging with a more positive attitude and showed more positive emotions. The expansion construction theory showed that positive emotions can effectively help individuals expand and construct their own emotional resources (41). Therefore, the subjective age of positive attitude strengthened the individual's resilience. When the level of resilience increased, the degree of loneliness decreased. Therefore, subjective age can affect loneliness through resilience.

### The independent mediating role of self-esteem between subjective age and loneliness

The results of the mediating effect test in this study showed that self-esteem was an important mediator between subjective age and loneliness; therefore, hypothesis 3 was verified. The research on the formation of subjective age showed that the more positive the subjective age of the old adults, the higher the level of self-esteem; the more negative the subjective age, the lower the level of self-esteem. Specifically, due to the information processing method of self-protection, the old adults drove self-enhancement through the prejudice of cognitive processing of self-information, so as to maintain or improve self-esteem and reduce the loss caused by age (42). Some studies have also confirmed that subjective age has a significantly positively predictive effect on self-esteem (43). The aging of the old adults led to the loss of the individual, but old adults still expect to keep the same level as before. Therefore, old adults maintained their self-worth and self-esteem by adjusting their self-cognition (reducing subjective age). Therefore, subjective age can significantly predict loneliness through self-esteem, and self-esteem was an important mediator between subjective age and loneliness.

### The mediating roles of resilience and self-esteem between subjective age and loneliness

The mediating effect test in this study also showed that subjective age could influence the loneliness of the old adults through the chain mediating effect of resilience and self-esteem; therefore, Hypothesis 4 was verified. The realization of this process was accompanied by a certain degree of testing of the old adults' own ability in the whole social field involved, so the old adults' ability was acknowledged to a certain extent in the process of helping others, thus generating a sense of self-affirmation and satisfaction, which were the key to the old adults' self-esteem. The effect of resilience was universal, which promoted an individual's ability to pay more attention to positive forms of self-evaluation (such as respect and approval) by others. This positive form of attention was conducive to improving the individual's self-evaluation, so it was the key to determine the level of self-esteem, that is, the level of resilience can affect the level of self-esteem, and a high level of resilience can promote the level of self-esteem. In addition, studies have shown that resilience can not only directly affect loneliness but also indirectly affect loneliness through influencing individual psychological state and neuroendocrine (44); therefore, resilience can affect loneliness through self-esteem.

## Implications

Through quantitative analysis, this study obtained important insights into reducing the loneliness of old adults. The old adults can not only maintain or reduce their subjective age but also ensure good resilience and maintain a high level of self-esteem to effectively reduce loneliness. This suggested that the problem of loneliness in old adults can be prevented and improved from three aspects. First, subjective age can be changed in various ways, such as a good sense of control, a positive attitude, and good daily habits (such as eating meals on time and regular exercise habits) that can change the subjective age and strengthen the old adults' sense of controlling over their subjective age. Although aging was inevitable, it was not terrible. Having a young subjective age, maintaining psychological vitality, and actively facing life were the keys to prevent loneliness on the way of aging. Second, this study showed that increasing the level of resilience can effectively prevent the loneliness of old adults. Therefore, individuals and families should not only pay attention to the material needs of the old adults but also pay attention to the spiritual needs of the old adults. Widowed, divorced, and unmarried seniors should also be supported to find a spouse. Nondiscrimination and empathy should be given to the old adults to establish stronger support. Third, with the development of modern society, the old adults have been knocked out by the times, resulting in certain social isolation and loneliness. Self-esteem was an effective means to regulate this isolation. Therefore, when old adults encountered fresh things, they should be patiently encouraged or taught to learn, so as not to let the old adults feel separated.

## Limitations and future directions

There are many aspects that can be improved in this study. First, this study adopted the method of self-report to measure subjective age, resilience, self-esteem, and loneliness, and some of the tests were carried out in public places such as parks and communities, which may have an effect on social approval. In the future, other ways (such as the evaluation of close people around the old adults) can be adapted to collect data to further improve the accuracy of the research results. Second, a cross-sectional method was used in this study to determine the relationship between variables through data analysis. In future research, a longitudinal research method can be adopted to explore the relationship between subjective age and loneliness development according to the developmental characteristics of subjective age, in order to determine the development law of subjective age and loneliness. In the future, we can also conduct research through experimental methods to observe the change in loneliness and determine the causal relationship between subject age and loneliness by manipulating the subjective age of the old adults. Third, subjective age and loneliness are complex concepts with multiple dimensions, and future research can explore the relationship between them from different perspectives. Fourth, this study is limited by the status of researchers, and before the questionnaire survey, it failed to comprehensively assess the physical health status of the old adults (e.g., whether there was a history of diabetes and physical dysfunction) and whether there was a history of mental illness. Therefore, in the future, this study can invite psychiatrists, doctors, and other professionals to evaluate the physical and mental health of the old adults and then conduct an investigation on the old adults according to the evaluation, so that the research results will be more objective and scientific. Besides, in this study, we have considered both loneliness and self-esteem as one-dimensional instead of multidimensional phenomena, and this may be a limit because this is inconsistent with the existing view. In the future, we can consider both loneliness and self-esteem as multidimensional phenomena.

## Data Availability Statement

The original contributions presented in the study are included in the article/supplementary material, further inquiries can be directed to the corresponding authors.

## Ethics Statement

The studies involving human participants were reviewed and approved by Ethics Committee of the Mental Health Service Center. The patients/participants provided their written informed consent to participate in this study.

## Author contributions

JX and BZ designed the study protocol. JX performed the statistical analysis and drafted the manuscript. CZ guided the statistical analysis and interpretation of the results and edited the final manuscript. ZY provided financial support, as well as guided the first draft of the manuscript and provided guidance on the overall design of the study and the revision of the manuscript. WZ and JW completed the literature review and participated in the study design and interpretation analysis. XH has provided guides in the process of revision. All authors contributed, read, and approved the submitted version.

## Conflict of interest

The authors declare that the research was conducted in the absence of any commercial or financial relationships that could be construed as a potential conflict of interest.

## Publisher's note

All claims expressed in this article are solely those of the authors and do not necessarily represent those of their affiliated organizations, or those of the publisher, the editors and the reviewers. Any product that may be evaluated in this article, or claim that may be made by its manufacturer, is not guaranteed or endorsed by the publisher.
